# Ecological habitat partitioning and feeding specialisations of coastal minke whales (*Balaenoptera acutorostrata*) using a recently designated MPA in northeast Scotland

**DOI:** 10.1371/journal.pone.0246617

**Published:** 2023-07-19

**Authors:** Kevin P. Robinson, Duncan A. I. MacDougall, Connor C. G. Bamford, William J. Brown, Ciaran J. Dolan, Rebecca Hall, Gary N. Haskins, Grace Russell, Theofilos Sidiropoulos, Texa M. C. Sim, Evgenia Spinou, Elice Stroud, Genevieve Williams, Ross M. Culloch

**Affiliations:** 1 Cetacean Research & Rescue Unit (CRRU), Banff, Scotland, United Kingdom; 2 Centre for Ecology & Conservation, University of Exeter, Cornwall, England, United Kingdom; 3 British Antarctic Survey, Cambridge, England, United Kingdom; 4 Scottish Association for Marine Science, Oban, Scotland, United Kingdom; 5 APEM Ltd, Stockport, England, United Kingdom; COISPA Tecnologia & Ricerca - Stazione Sperimentale per lo Studio delle Risorse del Mare, ITALY

## Abstract

In the design of protected areas for cetaceans, spatial maps rarely take account of the life-history and behaviour of protected species relevant to their spatial ambit, which may be important for their management. In this study, we examined the distribution and feeding behaviours of adult *versus* juvenile minke whales (*Balaenoptera acutorostrata*) from long-term studies in the Moray Firth in northeast Scotland, where a Marine Protected Area (MPA) has recently been designated. Data were collected during dedicated boat surveys between 2001 and 2022 inclusive, from which 784 encounters with 964 whales of confirmed age-class (471 juveniles and 493 adults) were recorded from 56,263 km of survey effort, resulting in 238 focal follows. Adults and juveniles were occasionally seen together, but their distributions were not statistically correlated, and GIS revealed spatial separation / habitat partitioning by age-class―with juveniles preferring shallower, inshore waters with sandy-gravel sediments, and adults preferring deeper, offshore waters with greater bathymetric slope. GAMs suggested that the partitioning between age-classes was predominantly based on the differing proximity of animals to the shore, with juveniles showing a preference for the gentlest seabed slopes, and both adults and juveniles showing a similar preference for sandy gravel sediment types. However, the GAMs only used sightings data with available survey effort (2008 to 2022) and excluded depth due to collinearity issues. Whilst adult minkes employed a range of “active” prey-entrapment specialisations, showing inter-individual variation and seasonal plasticity in their targeted prey, juveniles almost exclusively used “passive” (low energy) feeding methods targeting low-density patches of inshore prey. These findings corroborate the need to incorporate demographic and behavioural data into spatial models when identifying priority areas for protected cetacean species. Not all areas within an MPA have equal value for a population and a better knowledge of the spatial preferences of these whales within the designated Scottish MPAs, appointed for their protection, is considered vital for their conservation.

## Introduction

**Fig 1:**
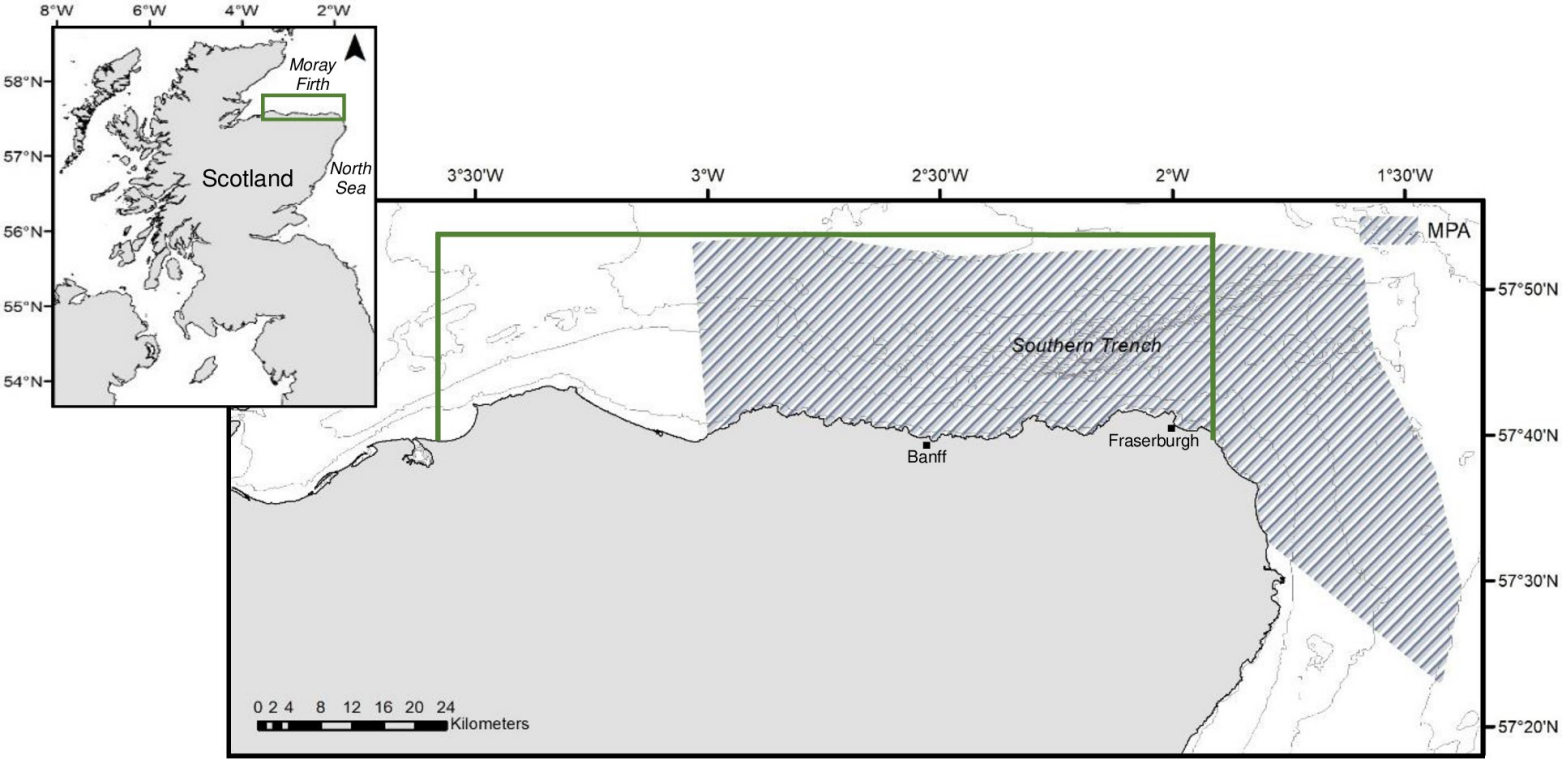
The location of the study area (green border) and the boundaries of the Southern Trench MPA (shaded) along the southern coastline of the outer Moray Firth in northeast Scotland.

The minke whale (*Balaenoptera acutorostrata* Lacépède) is the smallest and most abundant of the baleen whales in UK waters. Approximately 9,000 individuals occur in the North Sea [1], with most sightings inshore, in shelf waters less than 200 metres deep [2]. The highly productive waters of the Moray Firth in northeast Scotland (57° 41′ N, 2° 40′ W) attract above-average densities of minke whales relative to adjacent and wider North Sea waters [3], affording rich feeding grounds for these mammals during the summer and autumnal months [2, 4]. Accordingly, the Southern Trench, along the southern coast of the outer Moray Firth (Fig 1) was recently designated a Marine Protected Area (MPA) for these whales [5].

The defined boundaries of the Southern Trench MPA enclose shelf deeps (~200 metres in depth), core frontal systems and other geodiversity features such as burrowed mud [5]. The trench provides nursery areas for large numbers of juvenile fish [6], and the frontal zones concentrate nutrients and plankton attracting fish species and marine mammal predators alike [7].

Mapping high-density areas from distributional sightings data is a crucial first step in the design of area-based management for cetaceans [8]. However, spatial maps rarely take account of the life-history and behaviour of protected species relevant to their spatial ambit [9]. This may be important when modelling population trends or assessing the susceptibility of a species to anthropogenic threats, essential for conservation management. Robinson et al. [2] note the high percentage of juvenile minkes (comprising ~60% of all sightings) encountered in the Moray Firth region and their nearshore preference for sandeel (*Ammodytes marinus*) predicted habitat. Whilst the low energetic cost of swimming in these whales allows them to exploit environmental conditions over large spatial scales [10, 11], juvenile minkes are thought to be less efficient foragers than adults [2] and may be displaced from optimal feeding areas by their larger conspecifics, forcing them to forage elsewhere.

Dietary plasticity in baleen whales and the influence of eco-geographic variables upon the distribution of these marine predators and their prey is certainly well-documented [7, 12–14]. Variables such as ocean floor topography, water depth, sea bottom sediment, tidal fronts and water temperature, for example, evidently exert a strong influence on the distribution of minke whales and their prey across fine- and meso-scale levels [2, 15–19]. Indeed, foraging plasticity may enable these whales to change their feeding methods when faced with environmental and habitat changes.

In the following study, we investigated whether minke whales of different age-class exhibited differences in their spatial occurrence and feeding preferences in the coastal Moray Firth. The distribution of adults *versus* juveniles was examined with respect to the proximity of sightings to the shore, and the physiographic predictors water depth, benthic slope and sediment-type. Observational data were further incorporated to investigate the feeding methods and dietary preferences of minkes using these coastal waters and the variations in feeding strategies utilised by age class. The primary focus of this study was to identify priority areas within the newly designated MPA which might be biologically important for the species and informative to the adaptive management process.

## Materials and methods

### Data collection

Sightings data were collected during dedicated boat-based surveys within a 1,980 km^2^ area of the southern Moray Firth between May and October 2001 to 2022 inclusive. Dedicated surveys were carried out using rigid inflatable boats with a crew of two experienced and up to six additional trained observers searching for whales using a continuous scanning method. Only the initial sighting of each whale was used in the following investigation to avoid data pseudo-replication and autocorrelation, with focal follows lasting up to 30 mins. No focal follows were ever recorded from the same “marked” individual twice, either in the same or across successive years. After each whale encounter, the search effort was directed to previously un-surveyed areas to minimise repeated encounters of the same whales and to maximise the spatial coverage obtained during bi-weekly boat surveys. The surveys were largely conducted opportunistically, with selected routes chosen to maximise capture probabilities as informed from intra-seasonal wide-scale searches of the full study area.

Cues used to locate minke whales during surveys included the presence of feeding birds [4] in addition to direct observations of the animals themselves when travelling or surface feeding [2]. When a sighting was made, the time, immediate geographic position of the animal(s) (corrected for distance and bearing), behaviour (feeding/foraging or travelling) and age-class (adult/juvenile) of the whale(s) were recorded. Adult minkes were defined as large, dark coloured animals >6.5 metres in length, whilst juveniles were defined as lighter, olive-coloured animals <6.5 metres [20]. Sightings which could not be assigned to an age-class, due to the briefness of the encounter, evasiveness of the animal or poor lighting/sea conditions etc, were not included in the analysis (*n* = 186).

### Feeding behaviour

An ethogram was used to describe the surface feeding specialisations used by individual whales during observed predation events (Table 1). For each focal-follow, a minimum of six trained observers were tasked with tracking individual whales, providing 360° visual coverage around the survey vessel to ensure no surfaces / behaviours were missed. All follows were conducted off-survey effort (termed encounter effort), with boat distances maintained between 50 and 300 metres from the subject. Qualitative prey sampling was conducted opportunistically during individual follows/feeding events using a medium-mesh, extendable landing net (Aquascape Ltd, UK) for prey species identification. During each sampling event, *in situ* length measurements were recorded for a minimum of twenty prey items before returning the live-netted fish back to the sea.

**Table 1:** Ethogram detailing the surface feeding behaviours / prey entrapment methods employed by minke whales frequenting the coastal Moray Firth.

Behaviour	Description
***General***	
** Passive (bird-associated) feeding**	Whales exploit concentrations of baitfish compacted together at the water’s surface by flocks of feeding seabirds from above and schooling predatory fish from below
** Active feeding**	Whales actively corral the baitfish themselves, showing multidirectional surfacing followed by a variety of feeding strikes (aerial, surface or sub-surface lunges) in dorsal, ventral or lateral planes
***Active Feeding***	
**Pre-strike**	
** Head slap**	Whale lifts its head above the water and audibly slaps its chin down on the surface
** Depth-charge**	After surfacing and re-submersing, the whale exhales air forcefully underwater creating a cacophony of bubbles at the surface
**Strike**	
** Sub-surface lunge**	Whale strikes below the surface producing a wave of white water, but the body is not seen
** Surface lunge**	Whale breaks the surface of the water and the arcing body is visible above the surface
** Aerial lunge**	Whale lunges high out of the water and the entire head and body is viewed
** Plane of strike**	The whale strikes the prey in either dorsal, ventral or lateral (either right or left side down) planes
** Speed of strike**	The strike is either fast and powerful or slow and progressive
**Post-strike**	
** Roll**	The whale rolls to the right- or left-hand side post-striking

### Spatial analysis

A rectangular grid of the geographic study area was created using ArcGIS Desktop 10.6.1 (ESRI, USA), with each grid cell measuring 0.5 km^2^. All sightings data from May to October 2001 to 2022 inclusive were subsequently imported into ArcGIS to examine the spatial distribution of adult and juvenile minke whales with respect to the underlying eco-geographic variables water depth, bathymetric slope, proximity to shore and sediment type. Depth data were obtained from GEBCO (30-arc second dataset), whilst the sediment data were provided under licence from the British Geological Survey [21]. The slope layer was derived from the depth data using a custom GIS workflow, whilst proximities of sightings to shore were calculated using a geodesic Euclidean Distance tool. After a successive process of simplification and classification, all layers were converted to Boolean maps for generation of the respective values within each 0.5 km^2^ grid cell. Moran’s *I*-tests were run using the ‘ape’ package in R 3.1.2 (http://www.r-project.org) [22] to test for spatial autocorrelation of whale sightings per grid cell for each survey year.

### Data modelling

Presence-absence grids were created for the survey years 2009 to 2022 inclusive for which effort data (GPS survey tracks) were available. Survey track lines were created using the ‘Point to Line’ tool, with each track line being separated by a unique survey ID number. A column was created in the sightings data indicating minke whale presence, coded for by ‘1’, and a column created in the track line data indicating surveyed areas, coded for by ‘0’. These columns corresponded to used and available habitat respectively, as a binary probability of the occurrence response variable [e.g., 23]. Both vector layers were converted to raster grids using the ‘Point to Raster’ tool and the raster grid cell size was set as 3,000 (3 km^2^) to capture environmental variability and the distance range from the vessel that minke whales were typically detected within, whilst avoiding excessive extrapolation to areas which would not be well represented by this method. Derville et al. [24] considered waters 10 km either side of GPS tracks as surveyed to account for the maximum detection range of humpback whales from the vessel, but the detection range of sightings in this dataset was lower since the survey vessels used were much lower in height and since minke whales are considerably smaller and harder to detect than other rorqual whale species. In order to produce the presence-absence grids, the tracks and sightings raster grid values for each month-year combination were added together using the ‘Raster Calculator’ tool from the Spatial Analyst extension. Presence-absence grids were collated with fixed physiographic data layers (the explanatory variables) using the ‘Sample’ tool from the Spatial Analyst extension in ArcGIS.

Generalised additive models (GAMs) were subsequently used to examine the non-linear relationship between adult and juvenile minke whales and their habitat for this restricted data set, using logistic regression with a binomial response for the presence/absence of sightings per grid cell. The data were modelled using R 4.1.2 (R Core Team 2021). For each of the explanatory variables (water depth, bathymetric slope, proximity to shore and sediment type) collinearity was examined using the ‘pairs’ function in R. From these outputs, the maximum correlation observed was between proximity to shore and water depth, but since this correlation was not strong (0.49), all variables were subsequently retained for the following analysis. The slope variable was transformed using the ‘log’ function in R, corresponding to the natural logarithm, to reduce the influence of very high values of which few observations were made. GAM functions were accessed using the *mgcv* package in RStudio [25], with separate GAMs being performed for adult and juvenile whale sightings respectively.

Smooth functions for water depth, proximity to shore and the log-transformed slope were included in the initial models, with the maximum number of parameters (*k*) set at 4 for the depth smooth and 3 for the log(slope) smooth to avoid overfitting. Sediment type was further included as a parametric linear variable. Low-rank thin plate splines were chosen as the smoother type for all smooth terms. The restricted maximum likelihood (REML) for smoothness selection was also used to prevent overfitting.

A GAM check, carried out using the *gam*.*check*() function, indicated that *k* was set at an appropriate level for the water depth and log(slope) smooth terms (p > 0.05), but not for proximity to shore in the juvenile model. Accordingly, *k* was increased until the GAM check indicated an appropriate value had been chosen and the final value for *k* was 10. In the adult model, the GAM check indicated that all smooth terms had been assigned an appropriate value for *k*.

#### Model validation and variable selection

To assess whether the applied models met the assumptions of a GAM, model validation techniques were employed to examine the residuals. Plots of residuals *versus* explanatory variables were created and examined using the *binnedplot*() function from the *arm* package (version 1.12 to 2) [26]; to assess the homogeneity of residual variance. To check the assumptions of spatial independence in residuals, the mean residuals were plotted against the spatial variables by longitude and latitude.

The mean residuals from both juvenile and adult models were fairly similar across all sediment types and showed no obvious patterns against continuous explanatory variables, indicating the assumption of approximate residual variance homogeneity was adequately met by both models. There were no particularly strong patterns in residuals against latitude and longitude in either model, further indicating the residuals from both models approximately met the assumption of spatial independence.

Concurvity was assessed using the *concurvity*() function from the *mgcv* package looking at the worst case concurvity. This was found to be high (> 0.8) for the water depth and proximity to shore terms in both models, indicating a concurvity issue. Thus, depth was removed from both models, as the models retaining proximity to shore but removing water depth explained much more deviance than those retaining water depth and removing proximity to shore. Subsequent concurvity checks indicated no further concerns for either model. Removal of the depth smooth term from both models required reconsidering previous smooth term properties. GAM checks indicated that *k* = 4 was adequate for all smooth terms in the juvenile model (p > 0.05), but for the adult model *k* = 4 was adequate for the log(slope) (p > 0.05) but not for the proximity to shore smooth term (p < 0.05). As a result, *k* was set as 6 for the distance to coast smooth term. Inspection of residual plots for assessing model assumptions suggested the same conclusions as models including depth smooth terms.

## Results

### Spatial analysis

**Fig 2:**
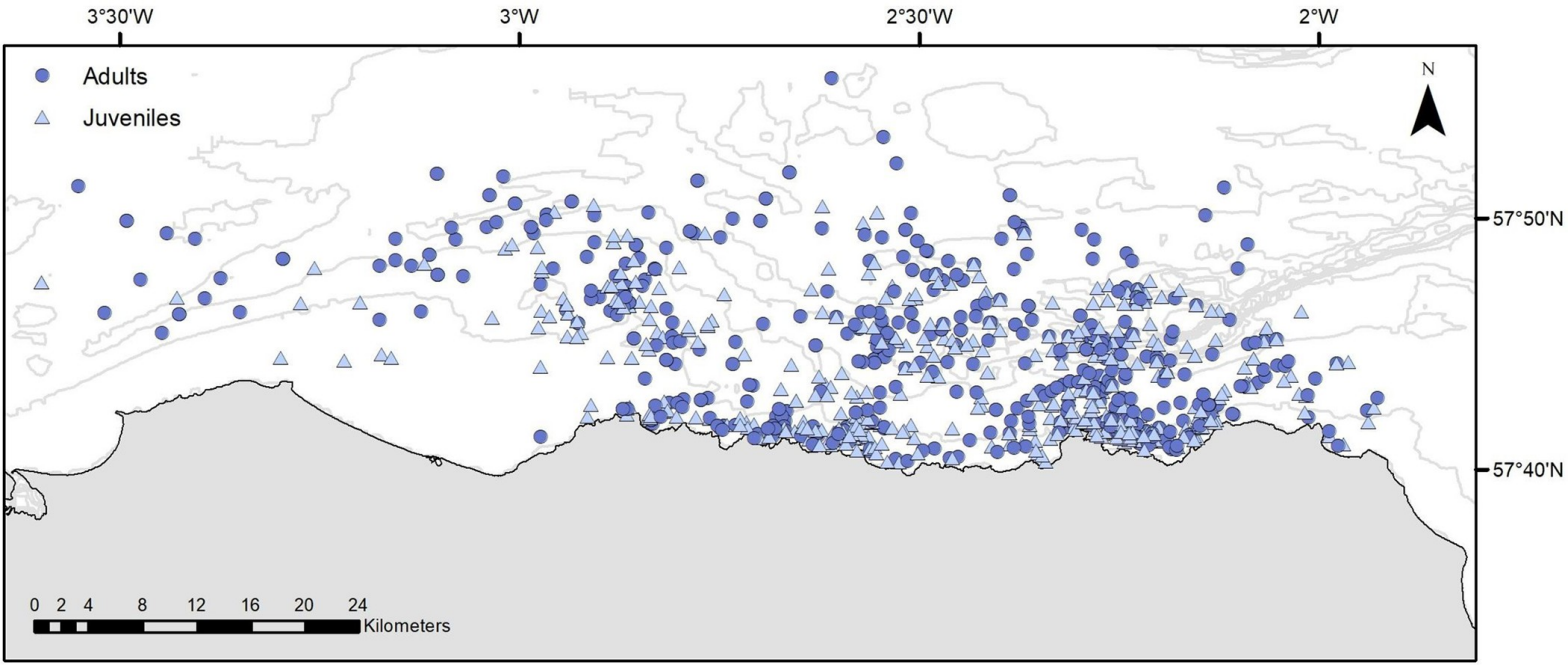
The spatial distribution of adult *versus* juvenile minke whale (*Balaenoptera acutorostrata*) sightings collated from long-term, dedicated boat surveys sightings in the Moray Firth study area between May and October 2001 to 2022 inclusive. A total of 56,263 kms of boat survey effort resulted in 964 sightings of confirmed age-class (471 juveniles and 493 adults).

**Fig 3:**
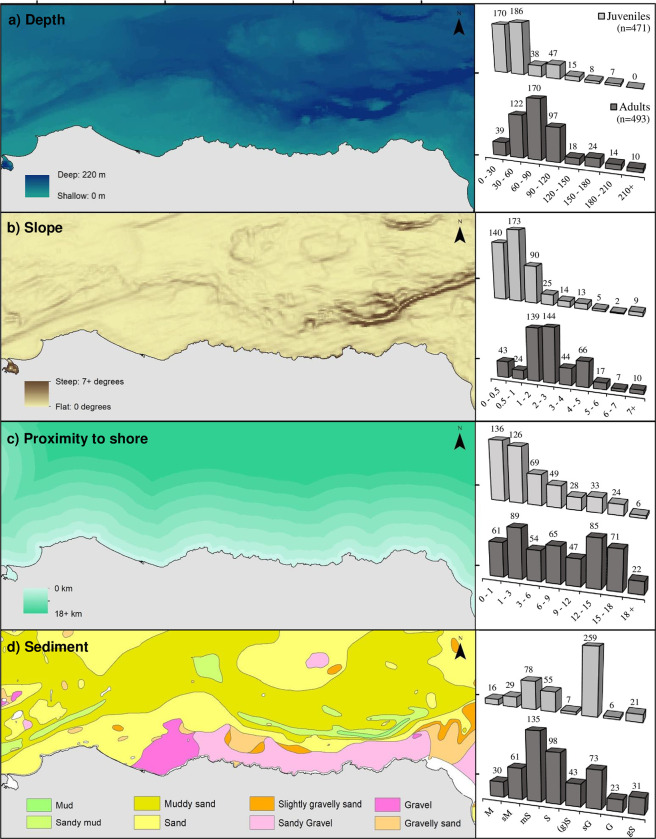
The spatial associations of adult and juvenile minke whales in the Moray Firth study area with respect to the eco-geographic variables (a) water depth, (b) bathymetric slope, (c) proximity to shore and (d) sea bottom sediment type.

The spatial distribution of all minke whale sightings of known age-class in the study area is illustrated in Fig 2. Between 2001 and 2022 inclusive, a total of 964 whales were recorded from 784 encounters. Feeding/foraging whales were predominantly encountered over travelling/resting whales (77% and 23% respectively), whilst juvenile animals were encountered almost as frequently as adults (471 juveniles c.f. 493 adults). Moran’s *I*-tests (p-values) revealed no autocorrelation in the sightings data across any of the survey years examined, and pairwise comparisons of sightings per grid cell further revealed that the spatial sightings of adults and juveniles were not statistically correlated. In addition, GIS resolutions inferred a strong association by juvenile minkes for shallow (mean depth = 24.2 ± 49.3 m), inshore waters (mean distance from coast = 2.66 km), with low benthic topography, whilst adult whales more typically occurred in deeper waters (mean depth = 54.2 ± 43.4 m), further from the coast (mean distance = 5.52 km), over areas of steeper bathymetric slope (Fig 3A–3C, Table 2). Sightings of juveniles were also strongly correlated with sandy gravel sediment (Spearman’s Rank Correlation r = 0.91, p < 0.001), whilst adults were predominantly associated with areas of sand and sandy mud (Spearman’s Rank Correlation r = 0.80, p < 0.05) (Fig 3D).

**Table 2:** The mean water depth, slope and proximity to shore of adult and juvenile minke whale sightings recorded in the outer Moray Firth between 2001 and 2022 inclusive (n = 964).

**Adults (*n* = 493)**	**Mean ± SD**	**Min**	**Max**
** ** *Depth (metres)*	54.2 ± 43.4	9.2	207.6
** ** *Slope (degrees)*	2.26 ± 1.82	0.03	10.02
** ** *Proximity to shore (km)*	5.52	0.88	26.70
**Juveniles (*n* = 471)**	**Mean ± SD**	**Min**	**Max**
** ** *Depth (metres)*	24.2 ± 49.3	4.5	202.6
** ** *Slope (degrees)*	0.88 ± 1.01	0.02	9.40
** ** *Proximity to shore (km)*	2.66	0.32	17.30

### Data modelling

**Fig 4:**
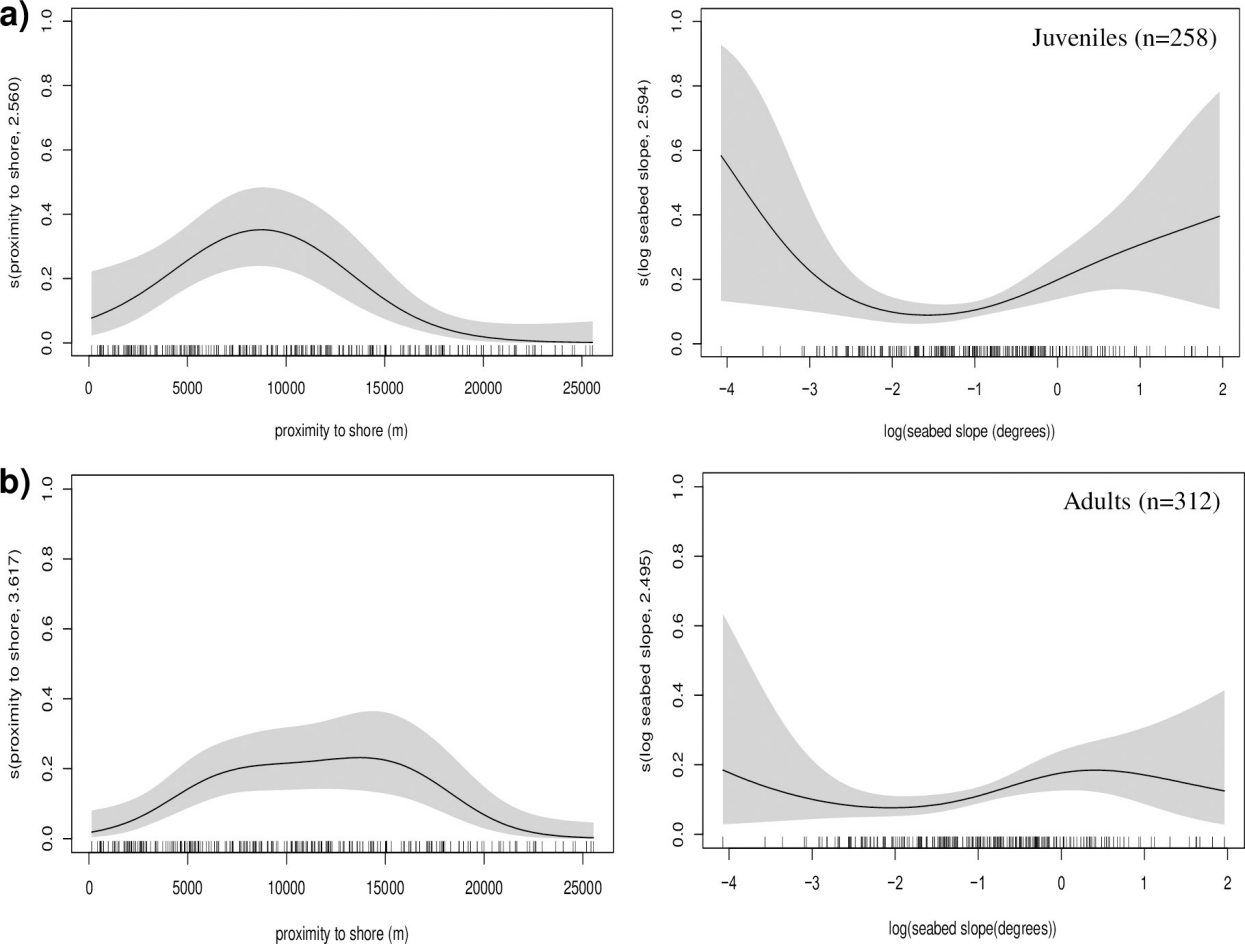
Partial effect plots displaying the component effect of (a) the smooth proximity to shore and the log seabed slope for (a) juvenile and (b) adult minke whales encountered in the Moray Firth study area between 2009 and 2022 inclusive (n = 570). Degrees of freedom are shown in parentheses on the y-axis label and the vertical lines above the x-axis show positions of the measured data points. The shaded areas represent 95% confidence intervals.

The selected models followed the form *n* ~ *s* (proximity to shore) + *s* (log(slope)) + (sediment type), with *n* denoting the probability of whale occurrence and *s* the smoother function for each covariate. The model explained 21.8% of the deviance for juvenile whales and 22% for adults. For juveniles, the probability of sightings was greater with closer proximity to the shore than in adults (Fig 4A). The smooth term for proximity to shore was subsequently found to be a significant predictor for whale occurrence (for juveniles χ^2^ = 21.72, edf = 2.6, p < 0.001, and for adults χ^2^ = 17.46, edf = 3.6, p < 0.05).

Juvenile occurrence probability was higher at low values of log(slope) (the gentlest slopes) and declined with log(slope) until values of approximately -1.6 (slope = 0.2 degrees), but then increased again slightly at high values of log(slope) corresponding to steeper slopes of up to 7.1 degrees (Fig 4B). For juveniles, the smooth function of log(slope) was estimated as a significant predictor of occurrence probability (χ^2^ = 8.89, edf = 2.6, p < 0.05). For adults, the probability of occurrence did not change greatly with log(slope) and the smooth function was found to be a non-significant predictor of occurrence probability in adults (χ^2^ = 5.84, edf = 2.5, p > 0.05). The probability of occurrence of both juvenile and adult whales over sandy gravel sediments was significantly greater than for all other sediment types (juveniles: Z = 2.48, p < 0.05; and adults (Z = 2.66, p < 0.05).

### Feeding behaviour

**Fig 5:**
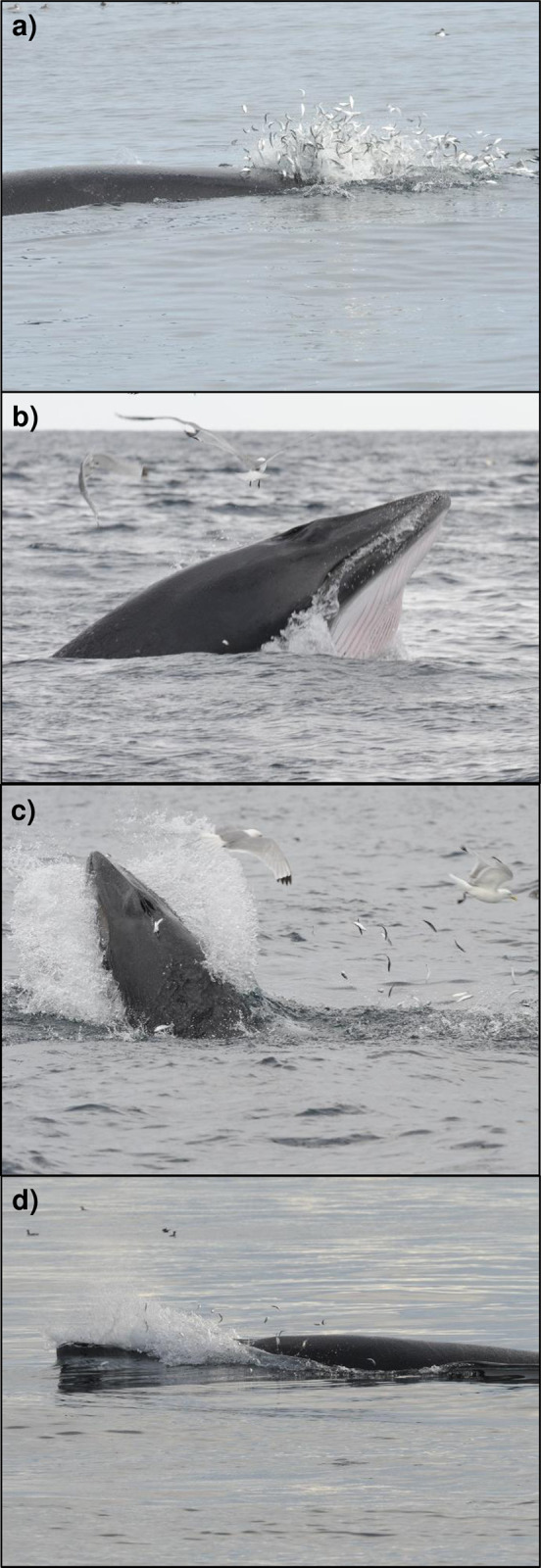
“Active” adult minke predation events upon juvenile herring (a and b) and upon pre-wintering sprat (c and d). Photographs: Kevin Robinson.

A total of 283 feeding strikes were recorded during 238 focal follows, with “passive” and “active” feeding behaviours being observed (Table 3). Active feeding was widely recorded in adults (Fig 5) but was only rarely documented in juveniles (in just 4% of the behaviours). In contrast, juveniles were, almost exclusively, observed using passive (bird-associated) feeding methods (Table 3). Whales of both age-classes were recorded engulfing prey using lateral and/or dorsal planes when striking. Lateral strikes were chiefly orientated right-side down, or with the whale rolling to the right post-strike, with just 10% of the animals performing left-sided manoeuvres (Table 3). Corralling behaviours such as head slapping and depth-charging (blow underwater after diving) were only ever used by adults, and aerial lunges (where the whale exited the water when feeding) were only performed in the absence of surface feeding seabirds. Forty-seven recognisable whales were recaptured during the study period on two or more separate sampling days. Of those individuals recaptured during different months in the same year (n = 11) or during different survey years (n = 14), on each occasion the same specific prey entrapment methods (orientation/type) were observed.

**Table 3:** Surface feeding specialisations recorded during individual focal follows (*n* = 238) of minke whales in the Moray Firth between 2001 and 2022.

	Adults	Juveniles	All
***Feeding type***			
** **Passive (bird-associated) feeding	16	160	176
** **Active feeding / corralling	116	7	123
** **TOTAL	132	167	299
***Pre-Strike Activity***			
** **Head slap	9	0	9
** **Depth-charge	21	0	21
***Type of Strike***			
** **Sub-surface	39	131	170
** **Surface	78	33	111
** **Aerial	15	3	18
***Plane of Strike***			
** **Dorsal	28	61	89
** **Ventral	7	0	7
** **Lateral	97	106	203
** ** *Right side down*	89	96	185
** ** *Left side down*	8	10	18
***Speed of Strike***			
** **Fast/powerful	112	130	242
** **Slow/progressive	20	37	57
***Post-Strike Roll***			
** **On the right-hand side	95	13	108
** **On the left-hand side	9	0	9

Prey items were collected from 95 feeding events between 2002 and 2017 (when focused prey sampling was conducted), Identified prey species comprised the lesser sandeel (*A*. *marinus*), Atlantic herring (*Clupea harengus*) and the European sprat (*Sprattus sprattus*) (Table 4). Juvenile whales preferentially targeted year 0–1 sandeels (between 86 and 118 mm in length). In adult whales, however, larger prey items, including year 0–3 sandeels (up to 163 mm in length), juvenile herring and pre-wintering sprat, were consistently sampled during observed feeding events (Table 4). Whilst sandeels were targeted by both adults and juveniles during all study months, May to October inclusive, juvenile herring were typically targeted only by adults from early July, whilst sprat were targeted by adults and juveniles from late August to October. The recorded seasonal changes in the proximities of animals to the shore with respect to the feeding methods employed and the prey species sampled for each age-class, are summarised in Table 5.

**Table 4:** Fish prey species sampled from individual feeding events (n = 95) of adult and juvenile minke whales between 2002 and 2017.

Age Class	——————Sandeel——————			——————Herring——————			——————Sprat——————		
	Mean length (mm)	Range (mm)	*n*	Mean Length (mm)	Range (mm)	*n*	Mean Length (mm)	Range (mm)	*n*
Adults (*n* = 52)	119	85–163	14	220	196–321	22	122	92–156	16
Juveniles (*n* = 43)	104	86–118	41	0	0	0	107	94–122	2

**Table 5:** Intra-seasonal changes in minke whale feeding techniques and proximity to shore from 95 feeding events observed between 2002 and 2017.

Interval	Age-class	Feeding methods		*Proximity to shore*			Prey species sampled		
		Passive	Active	*0–5 km*	*5–10 km*	*>10 km*	Sandeel	Herring	Sprat
** **May to Jun	Adults	9	30	***5 (10*.*4%)***	***29 (60*.*4%)***	***14 (29*.*2%)***	10	1	0
	Juveniles	74	0	***85 (68*.*5%)***	***36 (29%)***	***3 (2*.*5%)***	28	0	0
** **Jul to Aug	Adults	5	59	***44 (23*.*4%)***	***126 (67%)***	***18 (9*.*6%)***	2	17	3
	Juveniles	83	2	***136 (47*.*5%)***	***142 (49*.*7%)***	***8 (2*.*8%)***	11	0	0
** **Sep to Oct	Adults	2	12	***11 (11*.*7%)***	***59 (62*.*8%)***	***24 (25*.*5%)***	2	4	13
	Juveniles	3	4	***16 (47*.*1%)***	***17 (50%)***	***1 (2*.*9%)***	2	0	2

## Discussion

Occurrences of baleen whales on their feeding grounds are typically linked to environmental variables which influence the distribution of their prey [27, 28]. In the present study, using a correlative spatial analysis approach, proximity to shore and benthic slope were found to be significant predictors for the occurrence of adult minke whales, whilst sediment-type, proximity to shore and benthic slope were the most important predictors for juveniles. GAMs indicated that partitioning between age-classes was based predominantly on the proximity of animals to the shore and the benthic slope, with juveniles being preferentially occurring closer to the coastline than adults and showing a preference for sandy gravel sediments and gentle slopes, whilst adults showed a similar preference for sediment type but little preference for topographic slope. However, the models only used data from 2009 to 2022 (for which survey effort was available), and the effect of depth was not included due to collinearity with the proximity to shore variable, as the models retaining proximity to shore explained greater deviance than those retaining water depth. Since presence is a probabilistic function affected by species abundance and detectability [29], and assuming that the detectability of whales across all habitats was constant and that whales were equally detectable (as surveys were only conducted in sea states ≤ Beaufort 2), absences were subsequently associated with habitats in which abundance was low.

Juvenile whales were sighted in equally high numbers as adults within the study area, representing 49% of all sightings (as determined to age-class). Inevitably, juveniles utilising the study area may have been resighted on multiple occasions across separate survey days during the same year, and this may have biased these figures and potentially introduced pseudo-replication. However, since juvenile whales were typically absent of natural markings (i.e., dorsal fin nicks/tears), such as those more reliably used for individual identification of adults, it was not possible to correct for this bias. Nonetheless, not all adults had such markings that would allow recaptures over time, but where known recaptures of marked adults occurred in the database these data were used in the analyses. This would knowingly introduce pseudo-replication; however, identified recaptures were typically made months or years apart (i.e., recaptures were not close in time) and were relatively rare due to the high abundance of whales recorded in the study area. Consequently, as the object of the study was to examine the broad-scale habitat preferences of whales according to their age class, this was the preferred approach. Whilst juvenile and adult whales were occasionally seen foraging together in the study area, their distributions were also negatively correlated, further alluding to partitioning by age-class in the species. Indeed, Haug et al. [30] reported that, during their northward migration, minke whales show segregations by sex and size, with adult females and juveniles inhabiting more coastal areas and adult males tending to remain further offshore. In ecological systems, age- or sex-based differences may arise as a necessary consequence of body-size or development. such that partitioning might subsequently occur as a by-product of ontogeny [e.g., 31].

Based on opportunistic prey sampling, adult whales were observed targeting larger prey items than their juvenile counterparts. Juveniles were almost exclusively found to target year 0–1 sandeels (as confirmed from the sampling of targeted prey during feeding events), whilst adults showed greater dietary plasticity, with seasonal prey-switching between sandeels (year 0–3), herring and sprat―these three fish species together contributing up to 86% of the total fish biomass in this North Sea coastal region [32]. Sandeels are a short-lived, benthic fish, strongly associated with nearshore sandy-gravel sediments [33] to which the presence of juvenile whales were closely correlated. Conversely, herring and sprat are mid-water, shoaling species, occurring in deeper shelf waters [34, 35] where the adult whales were found to occur more typically. From June to August, juvenile herring seemed to be preferentially targeted by adult whales over sandeels, in response to their increasing availability in inshore waters as confirmed from preliminary environmental DNA sampling results (Robinson et al., unpublished data). However, from August to October, pre-wintering sprat were preferentially targeted over herring in response to the seasonal occurrence of these available prey. The complex schooling behaviour and strong predator avoidance shown by herring [35] may reduce the whales’ preference for herring when sprat are more widely accessible. However, predators naturally show heritable flexibility in their resource preferences when options are limited or when an alternative, high-valued resource becomes more widely available [36]. Within the Moray Firth study area in 2006, for example, following the EU-wide ban on the North Sea sandeel fishery [37], disproportionate numbers of both adult and juvenile minkes were sighted inshore, visibly profiting from the high densities of sandeel prey (K Robinson pers. observation). It is widely reported that minke whales respond to seasonal changes in the abundance of their prey [e.g., 11, 30], and this is assumed to occur when prey densities surpass a particular threshold that is energetically profitable for prey-switching to occur [2, 11]. This could conceivably explain the high interannual and intra-seasonal variability in resource selection noted in this and other UK studies of the species, and the apparent dietary plasticity shown by these generalist feeders [e.g., 11, 17, 19].

When hunting for prey, minke whales evidently employ a wide range of feeding techniques [e.g., 4, 38, 39]. In the present study, strategies included both “passive” (bird-associated) and “active” methods for prey entrapment, as first described by Hoelzel et al. [14]. Both adult and juvenile whales invariably pursued patchy resources within the study area, but the active feeding methods observed in adults were rarely, if ever, employed by juveniles. Instead, the juveniles typically utilised low energy (passive) prey entrapment methods, targeting small patches of ephemeral prey located close inshore [4]. In contrast, adult minkes exhibited a very broad range of individual specialisations, using a combination of mechanical behaviours to actively corral their prey, including head slapping and depth-charging (a forceful blow under water after diving) techniques that have also been described for the species in Canadian waters by Kuker et al. [40]. Interestingly, known (photo-identified) individuals using the Moray Firth study area utilised the same unique feeding specialisations during repeated encounters in different years (K Robinson pers. observation), perhaps alluding to an individually learned component of foraging [38] and resulting in the wide variety of feeding “styles” attributed to the species [e.g., 36]. In addition, the majority of feeding whales using the study area showed a clear preference for laterally-orientated feeding strikes (Table 4), showing a 90:10 right-handed bias, similar to the handedness index observed in humans [41]. A skewed ratio for directional lateral feeding has similarly been reported in humpback whales (*Megaptera novaeangliae*) [42] and blue whales (*Balaenoptera musculus*) [43], with individuals showing consistency in the orientation of their feeding strikes or rolls. One well-marked adult recaptured 6 times in the Moray Firth between 2006 and 2019, was only ever recorded using left-handed feeding manoeuvres, suggesting that basic brain lateralisation may be expressed in the same way in cetaceans as in other vertebrates [e.g., 44]. In this context, individual specialisation by these rorqual whales may yield positive benefits for their conservation by adding to the stability of populations [45] and their evolutionary diversification [46].

Given the spatial differences observed by age-class in the present study, the simplification of generalised habitat preferences universally described for this species, i.e., association with the 50-metre isobath, affinity for sandy-gravel sediments and preference for areas of steep topography [e.g., 2, 17, 19], may overlook ecologically-important niche separation reported herein. Clearly, not all parts of an MPA are of equal value for monitoring [8], and management objectives aiming to protect a species by targeting “average” resources pose a significant risk when intra-population variation exists due to demographic differences [e.g., 47]. The nearshore habitats utilised by juvenile minkes, for example, may harbour greater impacts from planned anthropogenic activities, such as the proposed export cable corridors and landfall sites for several consented wind farms, and associated increases in vessel traffic in these areas from harbours traditionally important for fishing activities. Indeed, one concern noted in the business and regulatory impact assessment for the MPA [48] is the risk of entanglement of whales with static fishing gear, such as creel pots, which are commonly used in this coastal region [49]. Therefore, based on the findings of the present study, juvenile whales would be at greater risk from entanglement, and hence in relation to the Conservation Objectives of the MPA, when assessing ‘favourable conditions’ against any potential impact, the importance of the differing eco-geographic variables to the relevant age classes should be considered. Furthermore, as preliminary photo-identification studies show that some whales utilising the Southern Trench MPA may remain in the region, or return over subsequent years (Robinson, unpublished data), there is a further risk of exposing the same individuals to anthropogenic stressors over time. Consequently, there is a need to integrate behavioural and demographic data into spatial models when identifying priority areas for protected cetacean species [e.g., 50], and to take these outputs into consideration when assessing the impacts of detrimental anthropogenic activities. Such assessments would also benefit from a better knowledge of the habitat preferences of these whales and the occurrence and site fidelity of individual animals. Continued surveying and monitoring of the MPA and neighbouring regions will be vital for the conservation and management of this species through better understanding of trends in presence, habitat use, feeding and foraging behaviour and niche segregation, from individual- to population-level.
